# Locally Advanced Pancreatic Head Cancer: Margin-Positive Resection or Bypass?

**DOI:** 10.5402/2012/513241

**Published:** 2012-06-18

**Authors:** Ulrich Friedrich Wellner, Frank Makowiec, Dirk Bausch, Jens Höppner, Olivia Sick, Ulrich Theodor Hopt, Tobias Keck

**Affiliations:** Department of General and Visceral Surgery, University of Freiburg, Hugstetter Street 55, 79106 Freiburg, Germany

## Abstract

Pancreatic cancer is a highly aggressive disease with poor survival. The only effective therapy offering long-term survival is complete surgical resection. In the setting of nonmetastatic disease, locally advanced tumors constitute a technical challenge to the surgeon and may result in margin-positive resection margins. Few studies have evaluated the implications of the latter in depth. The aim of this study was to compare the margin-positive situation to palliative bypass procedures and margin-negative resections in terms of perioperative and long-term outcome. By retrospective analysis of prospectively maintained data from 360 patients operated for pancreatic cancer at our institution, we provide evidence that margin-positive resection still yields a significant survival benefit over palliative bypass procedures. At the same time, perioperative severe morbidity and mortality are not significantly increased. Our observations suggest that pancreatic cancer should be resected whenever technically feasible, including, cases of locally advanced disease.

## 1. Introduction

The best treatment for pancreatic cancer without distant metastasis is surgical resection. Locally advanced tumors are incoherently defined in the current literature [1]. As portal venous resection (PVR) due to that tumor contact to the mesentericoportal axis is at present routinely performed in high-volume pancreatic centers [2, 3] a locally advanced tumor by surgical means is defined as a tumor with contact below 180 degrees to the mesenteric artery or celiac trunc. In these circumstances the surgeon is uncertain in the prediction of whether he can completely remove the tumor. The certainty to get tumor-free resection margins can in most of these cases only be determined after crossing a “point of no return” of the operation. Therefore, for locally advanced disease, the surgeon is confronted with the decision of whether to perform a pancreatoduodenectomy at the risk of positive surgical resection margins or to renounce resection and do a palliative bypass procedure instead.

A possible argument in favor of palliative bypass is reduced surgical trauma, as reduced postoperative morbidity and faster recovery are expected. This is also considered to allow the patient faster access to palliative therapy and longer and better quality of life in palliation. In addition, it might be argued that there is no benefit of resection over bypass in terms of survival if the resection margins turn out to be positive. The aim of this study was to analyze the perioperative and long-term outcome of surgical resection of pancreatic cancer with positive resection margins (R+) in comparison to palliative bypass procedures in locally advanced pancreatic tumors and also to put this into perspective with complete resection (R0).

## 2. Patients and Methods

From a prospectively maintained pancreatic surgery database at our institution, patients with pancreatic cancer operated from January 1994 to January 2011 were identified. Three groups were defined for analysis: complete margin negative (R0) resection, margin-positive resection (R+), and bypass procedures (BYP).

For histopathologic workup, the pancreatic, biliary, and retroperitoneal resection margins were labeled by the surgeon to allow for intensified workup of these areas in fresh frozen section analysis and definite histopathologic workup.

Operation and histopathological reports of the electronic patient records were rereviewed for data validation. Statistical analysis was performed with SPSS software version 17.0 (SPSS Inc., Chicago, IL, USA). The significance level was set to  *P* = 0.05. For dichotomous variables, the two-sided Fisher's exact test was used. The two-sided Mann-Whitney *U*-test or Kruskall-Wallis test was employed for statistical testing of ordinal and rational scale data. For survival analysis, the Kaplan-Meyer method with survival tables and survival plots was used for calculation and visualization of survival parameters and the Logrank test for statistical testing.

## 3. Results

### 3.1. Patients

From January 1994 to January 2011,  *n* = 367  patients with adenocarcinoma of the pancreas were operated at our institution. In seven cases the patients only received an exploratory laparotomy, which were excluded. In the majority (*n* = 243, 68% of total) of cases a pancreatoduodenectomy was performed, 71 (29%) resulted in margin-positive resections. In 117 cases (32% of total), a palliative bypass procedure was performed. Demographic data and comorbidities are shown in Table 1.

In preoperative evaluation of symptoms gastric outlet obstruction occurred more frequently in the BYP group (*n* = 40, 34%,  *P* < 0.05) and preoperative biliary drainage was most often performed in the R0 group (*n* = 116, 67%,  *P* < 0.05). Other significant differences involved a slightly higher rate of neoadjuvant therapy in the R+ group (*n* = 8, 11%,  *P* < 0.05) and a higher male-to female ratio in the bypass group (72 : 45,  *P* < 0.05). While resections were only incidentally performed in the setting of metastatic disease, the majority of patients in the BYP suffered from stage IV disease (*n* = 80, 68%). Other preoperative parameters as age, BMI, prevalence of diabetes, renal function, and bilirubin as a marker for biliary obstruction and cholestasis did not reveal significant differences between the groups.

### 3.2. Operations

With regard to the time necessary for the operative procedures (Table 1), pancreatoduodenectomy required in median twice the time of bypass procedures (445/470 min for R+/R0 versus 240 min for BYP,  *P* < 0.001). Resections with positive margins involved more frequently portal venous resections than R0 resections (R+ versus R0, 33% versus 48%,  *P* < 0.001), accompanied by an increase in operation time by 25 minutes in median and again indicating a higher rate of locally advanced tumors in this group.

### 3.3. Pathology

On histopathological examination (Table 1), tumors in the R+ group were significantly more locally advanced (T4 stage in R+/R0, 24%/4%,  *P* < 0.001) while lymph node metastases were not significantly more frequent (R+/R0, 79%/70%). 

### 3.4. Perioperative Outcome

Overall and surgical morbidities were highest in the R0 group and lowest in the BYP group (overall 53% versus 46%, surgical 35% versus 23%). For overall surgical morbidity, this showed to be statistically significant (*P* = 0.038) and translated to a lengthened hospital stay (R0/R+/BYP, 17/15/13 days,  *P* < 0.001). As shown in Table 2, this observation may be explained by the varying incidence of postoperative pancreatic fistula (POPF) in the different groups: POPF did, as expected, not occur in the BYP group, and the rate of POPF was significantly higher with R0 than with R+ resection (R0 versus R+, 11% versus 3%,  *P* = 0.044). There was also a significantly higher rate of postoperative bleeding with pancreatoduodenectomy compared to BYP (5%/9% for R0/R+ versus 1% for BYP,  *P* = 0.026). Furthermore, with R+ resection, more than half of the patients (54%) required perioperative blood transfusions, which was significantly higher than with R0 or BYP (44% and 6%,  *P* < 0.001). The incidence of delayed gastric emptying was similar in all three groups.

Despite the outlined increase in certain aspects of surgical morbidity with resection procedures, there was no obvious difference between R0, R+, or BYP regarding the most important perioperative outcome parameters severe morbidity, necessity of reoperation, or perioperative mortality.

### 3.5. Survival Analysis

The results of survival analysis are shown in Table 3. Survival data was available for  *n* = 359  patients. As control group, nonmetastatic disease treated by palliative bypass procedures (BYP-M0) was chosen, which had a median survival of 10 months and no patient was reported to survive three years. Margin-positive resections (R+) were divided into R1 (macroscopically negative but microscopically positive) and R2 (macroscopically positive margin).

**Figure 1:**
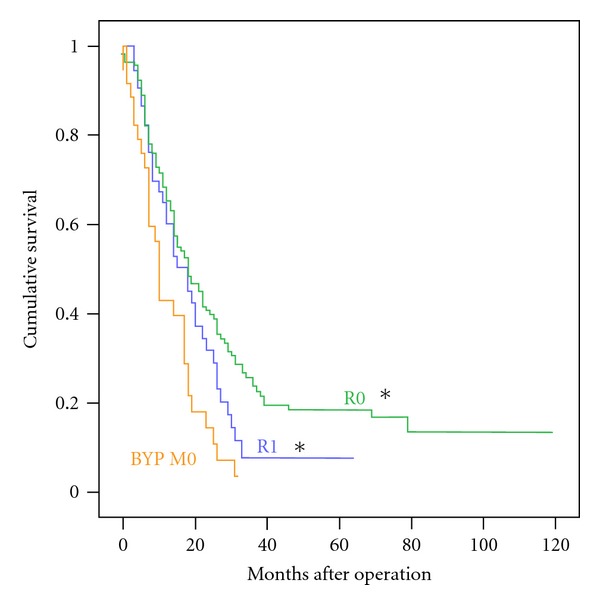
Survival after R1 resection. Kaplan-Meier Survival Function Plot. Asterisk denotes  *P* < 0.05  for Logrank test versus the control group BYP M0. BYP: palliative bypass procedure, M0: absence of distant metastasis at laparotomy, R0/R1: pancreatoduodenectomy with free/microscopically positive resection margins.

**Figure 2:**
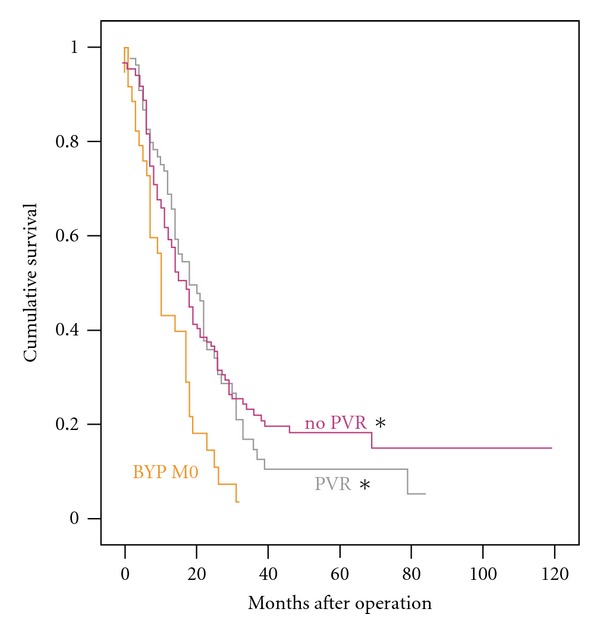
Survival after Pancreatoduodenectomy for Locally Advanced Tumors with Portal Venous Resection. Kaplan-Meier Survival Function Plot. Asterisk denotes  *P* < 0.05  for Logrank test versus the control group BYP M0. BYP: palliative bypass procedure, M0: absence of distant metastasis at laparotomy, and PVR: portal venous resection.

The R0 group had the best survival (median 18 months, 3-year survival 24%). As shown in Figure 1, patients after R1 resection still had a significantly better survival than the BYP-M0 group (median 18 months, 3-year survival 8%). Considering that locally advanced disease often involves portal venous tumor invasion, we also analyzed the survival for patients with portal venous resection (PVR). As shown in Figure 2, survival after resection was not significantly affected by PVR (median 18 months, 3-year survival 15%) which showed to yield significantly better survival than the control BYP-M0 group.

**Figure 3:**
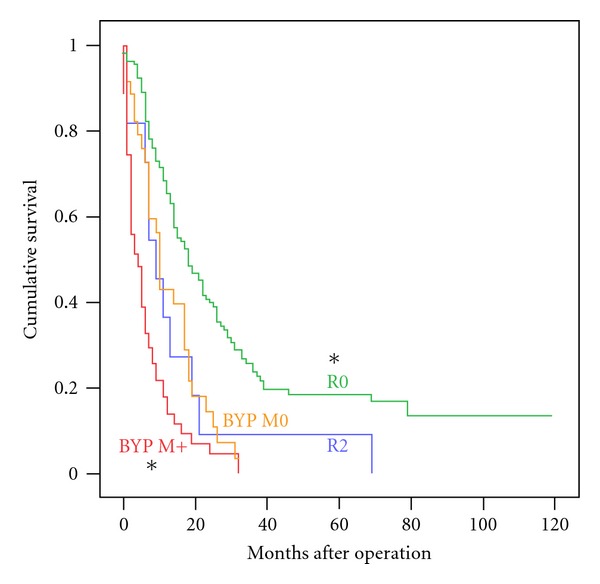
Survival after R2 resection and in metastatic pancreatic cancer. Kaplan-Meier Survival Function Plot. Asterisk denotes  *P* < 0.05  for Logrank test versus the control group BYP M0. BYP: palliative bypass procedure, M0/M+: absence/presence of distant metastasis at laparotomy, R0/R2: pancreatoduodenectomy with free/macroscopically positive resection margins.

As depicted in Figure 3, factors with significantly negative effect on survival were R2 situation and distant metastasis. Outcome after R2 resection (5% of all resections, median survival 9 months, 3-year survival 0%) was similar to the palliative BYP M0 group. In the setting of palliative bypass for metastatic disease to liver or peritoneum (BYP-M+), survival was significantly worse than any other group (median 4 months).

## 4. Discussion

On diagnosis of a pancreatic head tumor suspicious of malignancy and absence of metastatic disease, pancreatoduodenectomy is usually the treatment of choice. After dissection of the hepatoduodenal ligament and mobilization of the duodenum (Kocher maneuver), feasibility of macroscopic complete resection can be judged. However, quite often a tumor may be found to be “borderline resectable,” for example, in case of portal venous infiltration or invasion into the peripancreatic tissue. In this situation the surgeon is confronted with the decision to attempt radical resection at the risk of positive margins (R+) or to perform a palliative bypass procedure only. While it is clear that complete margin-negative (R0) resection will offer the best prognosis for the patient, the question whether there is a benefit from R+ resection over palliative bypass has only been addressed by few studies [4–8]. We aimed to compare the perioperative outcome and long-term survival of margin-positive (R+) pancreatoduodenectomy in comparison to palliative bypass, with margin-negative (R0) resection serving as a reference.

A major drawback of this study was that the decision whether to perform R+ resection or BYP was not randomized. However, no randomized study has been performed so far and probably never will be, as randomization to BYP would mean to deny the patient a chance of tumor resection in the setting of potentially resectable disease in this particular setting. Patients were grouped according to surgical procedure into R0, R+, and BYP. These groups were judged to be comparable in terms of demography and comorbidities, although there were distinct imbalances, mostly related to different stages of tumor progression. The incidence of distant metastasis and gastric outlet obstruction was highest in patients with BYP and the R+ resection group showed a higher rate of local T4 stage tumors and portal venous resections. Most of the palliative procedures in our collective were performed in the setting of metastatic disease, as this in general precludes pancreatoduodenectomy [9]. Nevertheless, these cases were included in the comparison of short-term perioperative outcome between resection and BYP.

Analysis of perioperative outcome showed that operation time, surgical morbidity, and hospital stay were significantly increased with pancreatoduodenectomy compared to BYP, mainly owing to occurrence of postoperative pancreatic fistula, which is virtually not possible in the case of BYP procedure. Prolonged operation time and hospital stay have also been noted in a previous study [8]. Interestingly, the POPF rate was lower with R+ resection, which may be attributed to advanced destruction of the exocrine pancreas with bigger tumors and hardened tissue consistency. A higher incidence of postoperative bleeding with pancreatoduodenectomy also contributed to the elevated surgical morbidity, which has also been observed by others [6]. Of note, more than half of the patients with R+ resection required perioperative blood transfusions, which can again be explained by more extended resection procedures in cases of locally advanced disease. However, in spite of increased surgical morbidity and most important, severe morbidity, rate of reoperation, and mortality were not increased with R0 or R+ pancreatoduodenectomy when compared to BYP. This is in line with previous observations [4–6, 8, 10].

For analysis of survival after resection, patients with palliative bypass procedures in the setting of nonmetastatic disease were defined as the control group. This distinction of metastatic disease was necessary in order to address the main issue whether R+ resection can offer a benefit over palliative bypass, as the resection is in general only performed in the absence of distant metastasis [9]. In the setting of metastatic disease, survival was very poor (median 4 months). It is questionable whether these patients benefit from surgical bypass, and limitation to interventional procedures has been suggested by other authors [11–14].

As expected, the best survival was achieved by R0 pancreatoduodenectomy, where about 1 of 4 patients survived 3 years. When resection margins were microscopically (R1) positive, patients still survived significantly longer than the palliative bypass control group. Regarding locally advanced disease, portal venous tumor infiltration necessitating portal venous en bloc resection did not negatively affect survival, which is in line with previous studies [2, 3]. Only in the rare case (<5% of resections) that macroscopic tumor had to be left (R2 situation) was survival statistically equal to the control group. This study is the first to compare the R2 subgroup separately from the R1 situation to palliative bypass for nonmetastatic disease.

Our observations draw attention to the ongoing discussion whether histopathologic examination of pancreatoduodenectomy specimen can correctly assess margin status [15, 16]. Surgical margin status in our study was not assessed according to recently published special workup protocols for pancreatoduodenectomy specimen [15, 17]. However, an intensified workup was performed at our institution because all resection margins, including the critical retroperitoneal margin, were routinely marked by the operating surgeon to be assessed microscopically by the pathologist.

**Figure 4:**
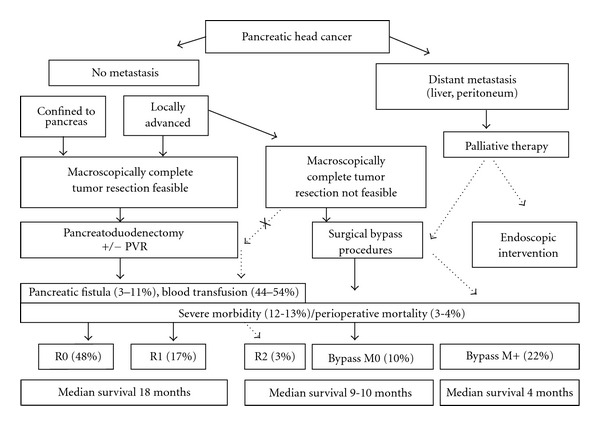
Flow scheme for the surgical treatment of pancreatic cancer. M0/M+: absence/presence of distant metastasis at laparotomy, R0/R2: pancreatoduodenectomy with free/macroscopically positive resection margins. Figures given from the present study.

In summary, perioperative morbidity is higher and hospital stay longer when pancreatoduodenectomy for periampullary cancer is compared to palliative bypass procedure, mainly as a result of pancreatic fistula and postoperative bleeding. However, this does not translate into increased severe complications or mortality. Coming back to the initial question, our findings suggest that radical resection of locally advanced pancreatic cancer should be preferred over palliative bypass and attempted whenever feasible.  Figure 4 summarizes our approach and findings of this study. This approach not only offers the chance of an R0 resection with the best survival. However, macroscopically incomplete resections should be avoided as they do not offer better survival. On the other hand, necessity of portal venous resection does not affect outcome. Even if resection margins turn out to be positive, our analysis demonstrates a survival benefit of approximately 8 months in favor of margin-positive pancreatoduodenectomy.

## Figures and Tables

**Table 1:** Patients, operations and pathology. *P* value given for two-sided Kruskal-Wallis and Fisher's exact test. Figures that cause a statistically significant difference are printed bold. R0/R+: pancreatoduodenectomy with free/positive resection margins, M0: absence of distant metastasis at laparotomy, M+: presence of distant or peritoneal metastasis at laparotomy, bypass: palliative bypass procedures, GE: gastroenterostomy, HE: hepaticoenterostomy, and double: GE and HE.

Parameter	Resection		Bypass	*P* value
	R0	R+		
*N*	172	71	117	—
Age (years, median)	66	65	67	0.211
Gender m : w	77 : 95	37 : 34	**72 : 45**	**0.020**
BMI (kg/m^2^, median)	24	25	23	0.166
Neoadjuvant CRx	6/4%	**8/11**%	4/3%	**0.046**
PreOP biliary drainage	**116/67**%	37/52%	63/54%	**0.022**
Gastric outlet obstruction	16/9%	4/6%	**40/34**%	<**0.001**
Diabetes mellitus	41/24%	25/35%	34/29%	0.188
Creatinine (mg/dL, median)	0.7	0.8	0.7	0.705
Bilirubin (mg/dL, median)	1.6	1.1	1.4	0.337
Resection procedures	Whipple 23/13% PPPD 149/87%	Whipple 14/20% PPPD 57/80%	—	0.305
Bypass procedures	—	—	Double 79/68% GE 30/26% HE 8/7%	—
Portal venous resection	57/33%	**34/48**%	——	<**0.001**
OP Time (Min, median)	445	470	**240**	<**0.001**
pT4 tumors	6/4%	**17/24**%	—	<**0.001**
Lymph node positive	119/70%	55/79%	—	**0.158**
Distant metastasis	3/2%	3/4%	**80/68**%	<**0.001**

**Table 2:** Perioperative outcome. *P* value given for two-sided Kruskal-Wallis and Fisher's exact test. Figures that cause a statistically significant difference are printed bold. ^1^Cases with perioperative mortality excluded. R0/R+: pancreatoduodenectomy with free/positive resection margins, M0: absence of distant metastasis at laparotomy, M+: presence of distant or peritoneal metastasis at laparotomy, bypass: palliative bypass procedures.

	R0 resection	R+ resection	Bypass	*P* value
*N*	172	71	117	—
Overall morbidity	91/53%	34/48%	54/46%	0.369
Surgical morbidity	**60/35**%	**23/32**%	**27/23**%	**0.038**
Severe morbidity	21/12%	9/13%	15/13%	0.867
POPF B/C	**19/11**%	2/3%	—	**0.044**
DGE B/C	21/12%	8/11%	12/10%	0.725
Postoperative bleeding	**9/5**%	**6/9**%	**1/1**%	**0.026**
RBC transfusion	**76/44**%	**38/54**%	7/6%	<**0.001**
Reoperation	15/9%	5/7%	11/9%	0.693
Overall mortality	7/4%	2/3%	7/6%	0.413
Surgical mortality	7/4%	2/3%	3/3%	0.758
Hospital days, median (range)^1^	**17 (9–62)**	**15 (9–61)**	**13 (4–80)**	<**0.001**

**Table 3:** Survival analysis. Survival function estimates by Kaplan-Meier method, *P* value given for Logrank test, R0/R1/R2: pancreatoduodenectomy with microscopically free/microscopically positive/macroscopically positive resection margins, PVR: pancreatoduodenectomy with portal venous resection, M0: absence of distant metastasis at laparotomy, M+: presence of distant or peritoneal metastasis at laparotomy, bypass: palliative bypass procedures.

	Resection group					Bypass group	
	R0	R1	R2	no PVR	PVR	M0	M+
*N*	171	60	11	151	91	37	80
% of total	48%	17%	3%	42%	25%	10%	22%
Median survival [months]	18	18	9	17	18	10	4
3-year survival	24%	8%	0%	22%	15%	<4%	0%
*P* value versus bypass M0	<0.001	0.041	0.923	0.003	0.002	—	0.002

## References

[1] Gillen S, Schuster T, Büschenfelde CMZ, Friess H, Kleeff J (2010). Preoperative/neoadjuvant therapy in pancreatic cancer: a systematic review and meta-analysis of response and resection percentages. *PLoS Medicine*.

[2] Yekebas EF, Bogoevski D, Cataldegirmen G (2008). En bloc vascular resection for locally advanced pancreatic malignancies infiltrating major blood vessels: perioperative outcome and long-term survival in 136 patients. *Annals of Surgery*.

[3] Riediger H, Makowiec F, Fischer E, Adam U, Hopt UT (2006). Postoperative morbidity and long-term survival after pancreaticoduodenectomy with superior mesenterico-portal vein resection. *Journal of Gastrointestinal Surgery*.

[4] Lavu H, Mascaro AA, Grenda DR (2009). Margin positive pancreaticoduodenectomy is superior to palliative bypass in locally advanced pancreatic ductal adenocarcinoma.. *Journal of Gastrointestinal Surgery*.

[5] Fusai G, Warnaar N, Sabin CA, Archibong S, Davidson BR (2008). Outcome of R1 resection in patients undergoing pancreatico-duodenectomy for pancreatic cancer. *European Journal of Surgical Oncology*.

[6] Kuhlmann K, De Castro S, Van Heek T (2006). Microscopically incomplete resection offers acceptable palliation in pancreatic cancer. *Surgery*.

[7] Lillemoe KD, Cameron JL, Hardacre JM (1999). Is prophylactic gastrojejunostomy indicated for unresectable periampullary cancer? A prospective randomized trial. *Annals of Surgery*.

[8] Lillemoe KD, Cameron JL, Yeo CJ (1996). Pancreaticoduodenectomy: does it have a role in the palliation of pancreatic cancer?. *Annals of Surgery*.

[9] Shrikhande SV, Kleeff J, Reiser C (2007). Pancreatic resection for M1 pancreatic ductal adenocarcinoma. *Annals of Surgical Oncology*.

[10] Kymionis GD, Konstadoulakis MM, Leandros E (1999). Effect of curative versus palliative surgical treatment for stage III pancreatic cancer patients. *Journal of the Royal College of Surgeons of Edinburgh*.

[11] Di Fronzo LA, Cymerman J, Egrari S, O’Connell TX (1999). Unresectable pancreatic carcinoma: correlating length of survival with choice of palliative bypass. *American Surgeon*.

[12] Scott EN, Garcea G, Doucas H, Steward WP, Dennison AR, Berry DP (2009). Surgical bypass vs. endoscopic stenting for pancreatic ductal adenocarcinoma. *Journal of the International Hepato Pancreato Biliary Association*.

[13] Schwarz A, Beger HG (2000). Biliary and gastric bypass or stenting in nonresectable periampullary cancer: analysis on the basis of controlled trials. *International Journal of Pancreatology*.

[14] Taylor MC, McLeod RS, Langer B (2000). Biliary stenting versus bypass surgery for the palliation of malignant distal bile duct obstruction: a meta-analysis. *Liver Transplantation*.

[15] Esposito I, Kleeff J, Bergmann F (2008). Most pancreatic cancer resections are R1 resections. *Annals of Surgical Oncology*.

[16] Jamieson NB, Foulis AK, Oien KA (2010). Positive mobilization margins alone do not influence survival following pancreatico-duodenectomy for pancreatic ductal adenocarcinoma. *Annals of Surgery*.

[17] Menon KV, Gomez D, Smith AM, Anthoney A, Verbeke CS (2009). Impact of margin status on survival following pancreatoduodenectomy for cancer: the Leeds Pathology Protocol (LEEPP). *Journal of the International Hepato Pancreato Biliary Association*.

